# Only Hematopoietic Stem and Progenitor Cells from Cord Blood Are Susceptible to Malignant Transformation by *MLL-AF4* Translocations

**DOI:** 10.3390/cancers12061487

**Published:** 2020-06-07

**Authors:** Kathy-Ann Secker, Lukas Bruns, Hildegard Keppeler, Johan Jeong, Thomas Hentrich, Julia M. Schulze-Hentrich, Barbara Mankel, Falko Fend, Dominik Schneidawind, Corina Schneidawind

**Affiliations:** 1 Department of Hematology, Oncology, Clinical Immunology and Rheumatology, University Hospital Tuebingen, 72076 Tuebingen, Germany; kathy-ann.secker@med.uni-tuebingen.de (K.-A.S.); lukas.bruns@student.uni-tuebingen.de (L.B.); hildegard.keppeler@med.uni-tuebingen.de (H.K.); dominik.schneidawind@med.uni-tuebingen.de (D.S.); 2 Synthego Corporation, Menlo Park, CA 94025, USA; jeong401@gmail.com; 3 Institute of Medical Genetics and Applied Genomics, University of Tuebingen, 72076 Tuebingen, Germany; thomas.hentrich@med.uni-tuebingen.de (T.H.); julia.schulze-hentrich@med.uni-tuebingen.de (J.M.S.-H.); 4 Institute of Pathology and Neuropathology, University of Tuebingen, 72076 Tuebingen, Germany; barbara.mankel@med.uni-tuebingen.de (B.M.); Falko.Fend@med.uni-tuebingen.de (F.F.)

**Keywords:** *KMT2A*-rearranged leukemia, CRISPR/Cas9, cell of origin

## Abstract

*Mixed lineage leukemia* (*MLL*) *(KMT2A)* rearrangements (*KMT2A*r) play a crucial role in leukemogenesis. Dependent on age, major differences exist regarding disease frequency, main fusion partners and prognosis. In infants, up to 80% of acute lymphoid leukemia (ALL) bear a *MLL* translocation and half of them are *t*(4;11), resulting in a poor prognosis. In contrast, in adults only 10% of acute myeloid leukemia (AML) bear *t*(9;11) with an intermediate prognosis. The reasons for these differences are poorly understood. Recently, we established an efficient CRISPR/Cas9-based *KMT2A*r model in hematopoietic stem and progenitor cells (HSPCs) derived from human cord blood (huCB) and faithfully mimicked the underlying biology of the disease. Here, we applied this model to HSPCs from adult bone marrow (huBM) to investigate the impact of the cell of origin and fusion partner on disease development. Both genome-edited infant and adult *KMT2A*r cells showed monoclonal outgrowth with an immature morphology, myelomonocytic phenotype and elevated *KMT2A*r target gene expression comparable to patient cells. Strikingly, all *KMT2A*r cells presented with indefinite growth potential except for *MLL-AF4* huBM cells ceasing proliferation after 80 days. We uncovered *FFAR2*, an epigenetic tumor suppressor, as potentially responsible for the inability of *MLL-AF4* to immortalize adult cells under myeloid conditions.

## 1. Introduction

The chromosomal translocations of the mixed lineage leukemia (*MLL*, *KMT2A*) gene are found in both acute myeloid leukemia (AML) and acute lymphoid leukemia (ALL) [1]. However, the frequency and the prognosis of *KMT2A* rearranged (*KMT2A*r) leukemia differ with age and the reasons for that are only partially understood. In infants, *KMT2A*r leukemia accounts for up to 80% of ALL with *AF4* as the main fusion partner and is associated with a particularly poor prognosis [2]. The responsible genetic alterations occur in utero in a cell of fetal origin [3]. Whereas in adults, the majority of *KMT2A*r leukemia is AML with *AF9* as the main fusion partner being associated with an intermediate prognosis. The translocation occurs most likely in hematopoietic stem or progenitor cells (HSPCs) derived from human bone marrow (huBM) possessing an inherent self-renewal capacity [1]. This is supported by the observation, that *KMT2A*r leukemias often co-express myeloid and lymphoid markers suggesting that the cell of origin has to be a HSPC qualified to express both lineage markers [4]. Further evidence is supplied by the clinical observation that *KMT2A*r ALL following CD19-targeted therapy can represent as AML in relapse [5]. Similarly, only HSPCs live long enough that additional genetic alterations, that may be required for *KMT2A*r leukemogenesis, can accumulate in this cell type and its progeny. Whereas adult *KMT2A*r leukemia often occurs in pretreated patients as secondary acute leukemia and additional cooperating mutations are mostly required for full leukemia transformation, infant *KMT2A*r leukemias develop as de novo leukemia bearing only very few additional mutations [6,7,8]. Thus, the consequences of genetic changes in HSPCs derived from infant and adults leading to leukemia development are distinct in both cell types. In humanized mouse models, Horton et al. could show that human cord blood (huCB) cells were more susceptible for a retroviral *MLL-AF9* immortalization, whereas retrovirally transduced huBM cells failed to immortalize in vitro and did not develop leukemia in vivo [9]. Similarly, by using mouse cells and an *MLL-ENL* fusion transcript Okeyo-Owuor et al. could demonstrate that the efficiency of *MLL-ENL*-driven AML changes with age with a peak shortly after birth [10]. In addition, the respective fusion partner has potential influence on leukemia development, as we and others could demonstrate its important role on the resultant phenotype: *ENL* exclusively led to ALL, whereas *AF9* presented as AML, ALL or mixed phenotype in mouse xenograft models [11,12]. Moreover, the microenvironment plays a dominant role in instructing lineage fate [13]. In summary, these observations imply that crucial differences in leukemia development exist dependent on the fusion partner, the microenvironment and finally the cell of origin, in which the mutation develops. However, until now the performed studies were mainly based on artificial systems solely utilizing mouse cells or retroviral transduced oncogenes with unknown effects for the resultant human leukemias. In this study, we used CRISPR/Cas9 to introduce translocations of the *MLL* and *AF4* or *AF9* genes under physiologic promotors in both huCB and huBM cells, faithfully mimicking the patient nature of the disease.

## 2. Results

### 2.1. CRISPR/Cas9 Demonstrates High Cutting Efficiencies and Induces t(9;11) and t(4;11) Chromosomal Translocations in Human HSPCs Derived from huBM

**Figure 1:**
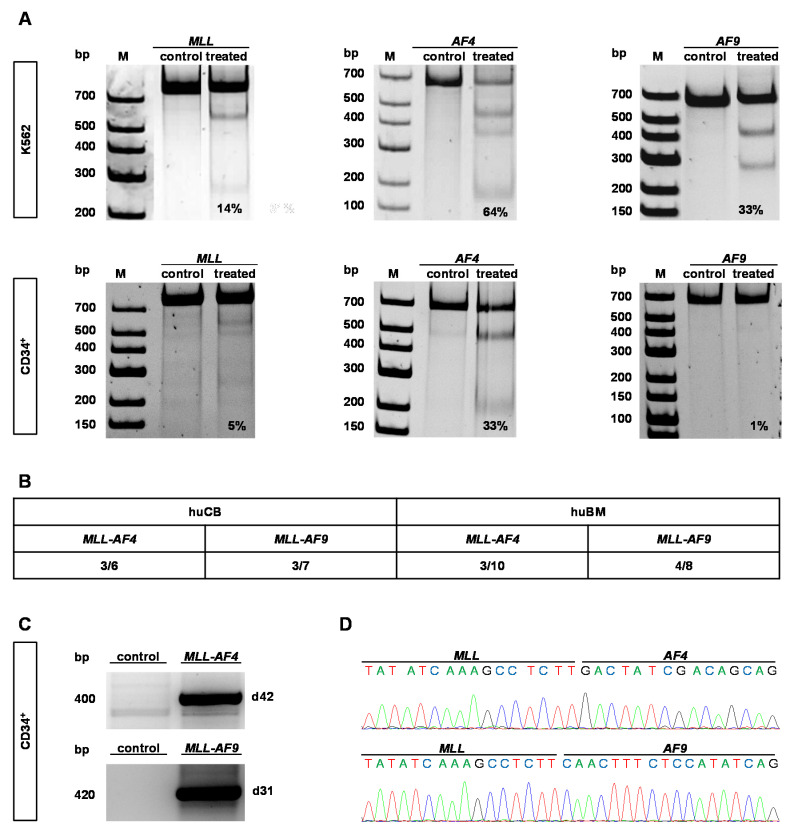
CRISPR/Cas9 induces specific double-strand DNA breaks within the *mixed lineage leukemia* (*MLL*), *AF4* and *AF9* genes and pairwise application leads to translocations in hematopoietic stem and progenitor cells (HSPCs) derived from adult bone marrow (huBM). (**A**) Gel images show representative results of T7 endonuclease assays performed on genomic DNA isolated from K562 cells (upper row) or HSPCs of huBM (CD34^+^) (lower row) nucleofected with single guide (sg)RNAs for the *MLL*, *AF4* or *AF9* gene and Cas9 protein. Cas9 alone was used as control. Digested PCR products represent the presence of strand mismatches resulting from indels that are generated by non-homologous end joining (NHEJ) repair of double-strand breaks (DSBs) and were quantified with ImageJ. Resulting cutting efficiencies are displayed. (**B**) Summarized data of positive translocation products of all performed experiments analyzed via PCR are shown. (**C**) Representative positive PCR products of genomic DNA isolated from CD34^+^ huBM nucleofected with *MLL* and *AF4* or *AF9* sgRNAs and Cas9 protein or Cas9 alone (control) at indicated time points are shown. (**D**) Sanger sequencing results of PCR products displayed in (**C**) are shown.

Previously, we were able to introduce *MLL-AF4* and *-AF9* chromosomal translocations based on patient sequences in HSPCs (CD34^+^) derived from huCB in high frequency [1,14]. To translate our results in an adult system, we used HSPCs derived from huBM in comparison to huCB to evaluate whether the cell of origin and/or the fusion partner influence leukemia initiation. By nucleofecting plasmid- and virus-free single guide (sg) RNAs for the genes *MLL*, *AF4* or *AF9* with Cas9 protein in K562 cell line as proof-of-principle and in HSPCs derived from huBM, respectively, we demonstrated successful cutting efficiencies in both cell types (Figure 1A). To induce *t*(9;11) and *t*(4;11) translocations in adult HSPCs, we isolated CD34^+^ cells and nucleofected them using Cas9 protein and sgRNAs targeting *MLL* and *AF4* or *AF9*, respectively. Cas9 alone was used as control. Following nucleofection, the cells were maintained in liquid culture supplemented with cytokines and chemokines optimized for growth of *KMT2A*r cells [15]. PCR analyses of genomic DNA revealed signals of *MLL-AF4* translocations in three out of 10 and *MLL-AF9* translocations in four out of eight performed experiments with different donors, demonstrating an easy translation of our previously used CRSIPR/Cas9-system to adult cells (Figure 1B). Sanger sequencing revealed specific fusion sequences comparable to our huCB approach (Figure 1C,D) [14]. These results demonstrate that we were able to induce *MLL* translocations with high frequency in HSPCs derived from huBM by using genome engineering.

### 2.2. Engineered Adult KMT2Ar Cells Are Characterized by KMT2Ar-Typical Gene Expression, Phenotype and Morphology

**Figure 2:**
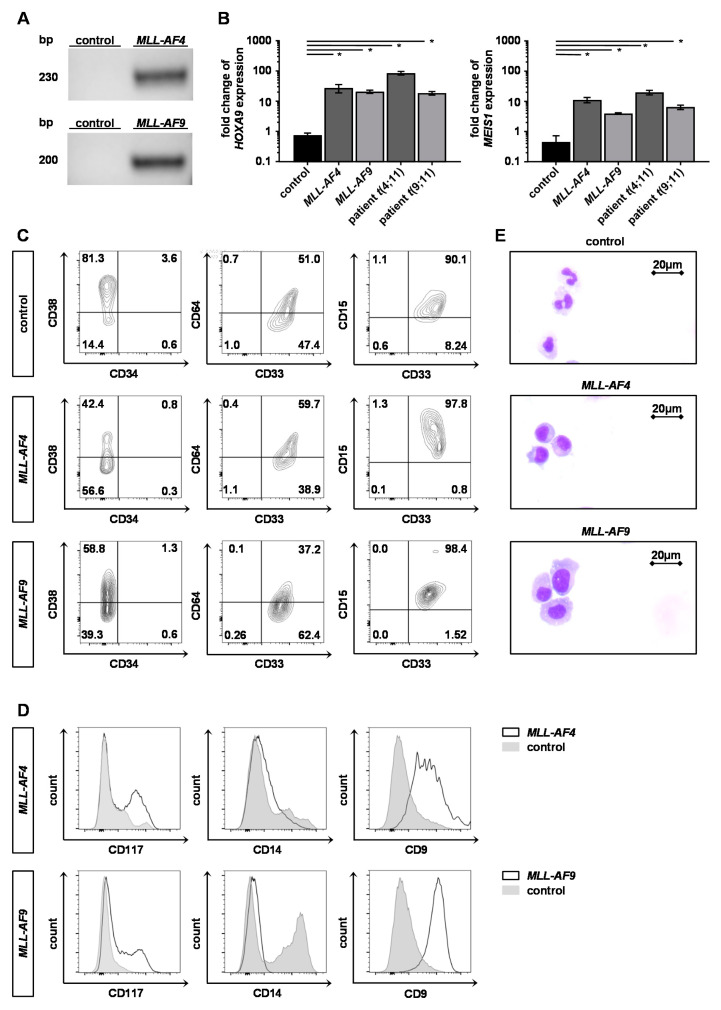
*KMT2A*r huBM cells reveal a patient-like gene expression profile and present with typical *KMT2A*r myelomonocytic immunophenotype. (**A**) mRNA was isolated from *MLL-AF4*/*-AF9* huBM cells or control cells (CD34^+^ huBM cells nucleofected with Cas9 alone) and fusion transcripts were identified by RT-PCR. (**B**) Expression of *KMT2A*r-specific target genes *MEIS1* and *HOXA9* was measured by qPCR. *MLL-AF4* and *MLL-AF9* huBM cells were normalized to control cells (CD34^+^ huBM cells nucleofected with Cas9 alone) and compared to patient cells harboring *t*(4;11)(q21;q23) or *t*(9;11)(p22;q23), respectively. Experiment was performed in biological duplicates (*n =* 2) and horizontal bars represent the mean. Student’s *t* test was used: * *p* < 0.05. Error bars indicate standard deviation (SD). (**C**) Representative contour plots of flow cytometry analyses of *KMT2A*r huBM and control cells regarding myelomonocytic markers CD15, CD33 and CD64 as well as the expression of CD34 and CD38 after reaching purity are shown. (**D**) *KMT2A*r huBM cells (black line) present with higher expression levels of immaturity marker CD117, lower expression levels of differentiation marker CD14 and higher expression of known *KMT2A*r surface marker CD9 compared to control cells (gray shading) [15,16]. (**E**) Representative morphologies of *KMT2A*r huBM and control cells are shown to display less cell differentiation and cell death of *KMT2A*r cells. Scale bars define 20 µm.

To characterize the engineered *KMT2A*r huBM cells, we performed RT-PCR to determine the functional expression on RNA level revealing both *MLL-AF4* and *-AF9* fusion transcripts (Figure 2A). Furthermore, we assessed the expression of common *KMT2A*r-specific target genes like *MEIS1* and *HOXA9* in *KMT2A*r huBM cells that were comparably high to the respective expression levels of patient-derived *KMT2A*r cells (Figure 2B).

Blast cells from *KMT2A*r leukemia patients typically display a myelomonocytic phenotype and no expression of CD34 in contrast to the most non-*KMT2A*r leukemia patients [16,17]. Likewise, the genome-engineered *KMT2A*r huBM cells in our model expressed myelomonocytic markers like CD64, CD33 and CD15 and lacked CD34 expression on their surface (Figure 2C). Interestingly, despite prolonged culture time of several weeks the *KMT2A*r huBM cells expressed almost no CD14 as marker of differentiation, but CD117 as marker of immaturity, whereas control cells differentiated upon time in culture (Figure 2D). Importantly, as further validation of our adult *KMT2A*r model, the genome engineered *KMT2A*r huBM cells expressed CD9 as typical *KMT2A*r leukemic surface marker (Figure 2D) [15,16].

To further characterize the *KMT2A*r huBM cells, we performed cytospins followed by May–Gruenwald–Giemsa staining that revealed an immature morphology of the *KMT2A*r huBM cells while the control cells presented a macrophage-like morphology and karyopyknosis indicating ongoing apoptosis (Figure 2E). These results demonstrate that using CRISPR/Cas9 to induce *MLL* translocations in HSPCs derived from huBM leads to expression of the fusion transcript, upregulation of *KMT2A*r-specific target genes, a myelomonocytic phenotype and immature morphology hereby authentically mimicking *KMT2A*r leukemia; therefore, this model can be used as a reliable patient-derived in vitro model.

### 2.3. MLL-AF9 Can Immortalize Neonatal and Adult Cells, Whereas MLL-AF4 Only Immortalizes Neonatal Cells

**Figure 3:**
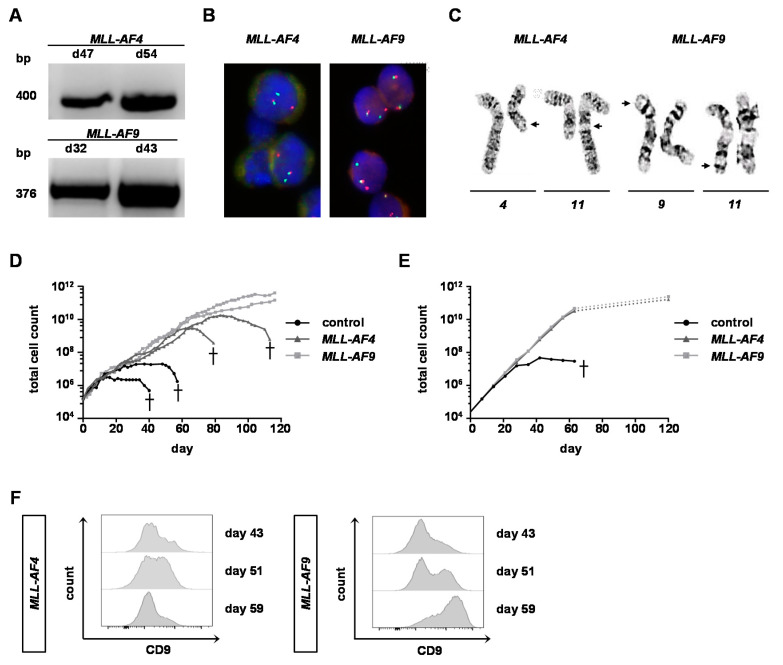
*MLL-AF4* translocation only immortalizes HSPCs of neonatal origin. (**A**) Representative semi-quantitative PCR of genomic DNA (100 ng) isolated from *KMT2A*r huBM cells over time of culture is displayed showing increasing *MLL-AF4/-AF9* PCR products. (**B**) Fluorescence in situ hybridization (FISH) analyses of *KMT2A*r huBM cells were performed after reaching strong PCR products (on day ~50 of culture). Manual inspection of 100 cells demonstrates 100% purity of the *KMT2A*r huBM cells. (**C**) Representative chromosomal translocations are displayed after G-banding analyses. Arrows indicate the reciprocal breaks. (**D**,**E**) Individual proliferation curves for the respective huBM (**D**) and human cord blood (huCB) (**E**) donor cells with or without *MLL* translocation are shown. (**F**) Representative flow cytometry analysis of CD9 expression on *KMT2A*r cells over time is displayed.

Following nucleofection with the respective sgRNAs targeting *MLL* and *AF4/AF9* and Cas9 protein, the huBM cells were kept in culture and monitored over time by PCR to detect the fusion gene. Both *MLL-AF4* and -*AF9* PCR products were detected with increasing signal intensity over time (Figure 3A). To quantify the percentage of cells with translocations, fluorescence in situ hybridization (FISH) and G-banding analyses were performed. On day 45 (*MLL-AF4*) and day 52 (*MLL-AF9*) of liquid culture, an *MLL* break-apart probe detected *MLL* translocations in 100% of huBM cells and G-banding analysis demonstrated the presence of both derivative chromosomes resulting in reciprocal *t*(4;11) and *t*(9;11) translocations (Figure 3B,C). Importantly, *MLL-AF9* translocations led to unlimited in vitro growth of both the genome engineered huBM and huCB cells (Figure 3D,E). However, *MLL-AF4* only immortalized huCB cells, whereas *MLL-AF4* huBM cells ceased proliferation around 60–90 days and finally underwent apoptosis in all performed experiments despite a stable *MLL-AF4* fusion expression (Figure 3A,D). Interestingly, some days in advance, the *MLL-AF4* huBM cells already downregulated CD9 expression on their surface indicating the incomplete transformation of the cells, whereas *MLL-AF9* cells demonstrated an increase of CD9 over time (Figure 3F). Collectively, these data indicate that *MLL-AF9* can immortalize CD34^+^ cells of both neonatal as well as adult origin in vitro, whereas *MLL-AF4* can solely immortalize cells of neonatal origin comparable to *KMT2A*r patient leukemia, as the portion of *MLL-AF4* leukemia in adults is very rare. These results indicate an important role of the cell of origin dependent on the expression of the respective fusion transcript for leukemia development.

### 2.4. Identification of Common KMT2Ar Target Genes and Uncovering of FFAR2 as Possible Intrinsic Factor Responsible for Cell Transformation

**Figure 4:**
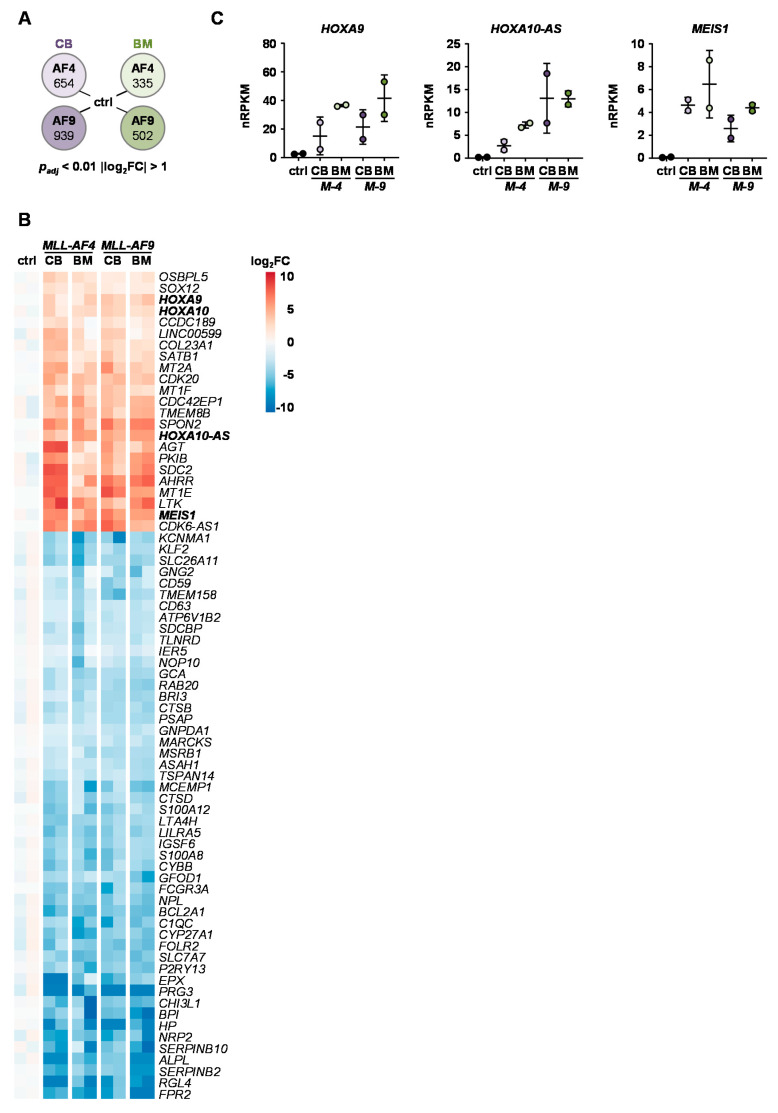
*KMT2A*r-specific target genes are overexpressed in *KMT2A*r cells derived from both huCB and huBM. RNA-seq was performed with *MLL-AF4* and -*AF9* cells derived from huBM and huCB and control cells (*n* = 2 for each group). (**A**) Total numbers of differentially expressed genes (DEGs) are displayed comparing *KMT2A*r cells of each origin to control cells. (**B**) The 73 DEGs concordantly changed in all groups following *MLL* translocation are displayed representing a common *KMT2A*r gene signature. (**C**) RNA-seq revealed *KMT2A*r-specific target genes *HOXA9*, *HOXA10*-*AS* and *MEIS1* to be differentially expressed in *KMT2A*r cells compared to control cells. Normalized reads per kilobase million (nRPKMs) are plotted as individual data points with the mean. Error bars indicate SD.

We performed RNA sequencing (RNA-seq) of the *MLL-AF4/-AF9* huCB and huBM cells and control cells to shed light on the transformation potential of *MLL-AF4* and *-AF9* translocations in the different cell types. Genes changed specifically in adult huBM cells upon *MLL-AF4* expression. This may provide insight into the mechanisms responsible for the observed phenomenon that solely huCB cells but not huBM cells were immortalized by the expression of the *MLL-AF4* fusion transcript. We identified 335 and 502 differentially expressed genes (DEGs) in huBM cells upon *MLL-AF4* and *MLL-AF9* expression, respectively. Regarding huCB, we detected 654 DEGs upon *MLL-AF4* and 939 DEGs upon *MLL-AF9* expression, respectively (Figure 4A). Of these genes, 73 were concordantly changed in all groups following *MLL* translocation and therefore represent a common *KMT2A*r gene signature that was irrespective of tissue source (Figure 4B). Interestingly, this signature comprised the upregulation of classical *KMT2A*r target genes like *HOXA9*, *10*, *10-AS* and *MEIS1* (Figure 4C). These data confirm that both our CRISPR/Cas9-*KMT2A*r models are physiologically relevant and that the inability of *MLL-AF4* to immortalize adult cells is not explained by absence of *KMT2A*r-induced expression of these common target genes.

**Figure 5:**
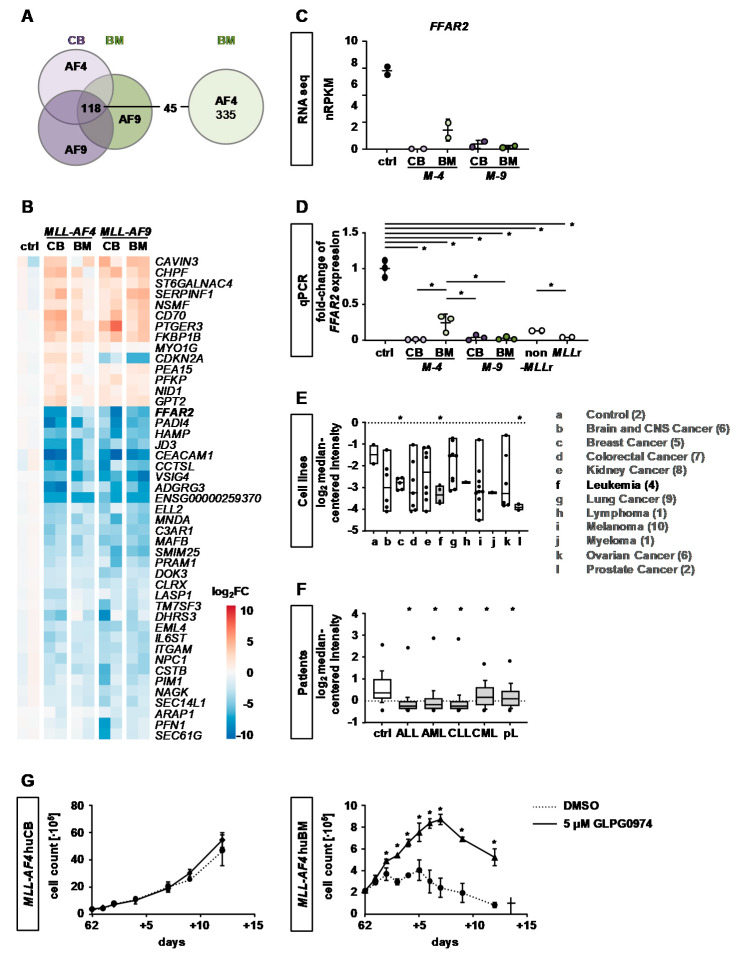
*FFAR2* as potential key player in the *KMT2A*r leukemogenesis. RNA-seq was performed with *MLL-AF4* and -*AF9* cells derived from huBM and huCB and control cells (*n* = 2 for each group). (**A**) DEGs of *MLL-AF4* huBM cells were compared to DEGs of all other *KMT2A*r cells and 45 genes were identified as differentially expressed. (**B**) Heatmap of the DEGs described in (**A**) is displayed. (**C**) RNA-seq revealed *FFAR2* to be differentially expressed when comparing *MLL-AF4* huBM DEGs to other *KMT2A*r cells. Normalized reads per kilobase million (nRPKMs) are plotted as individual data points with the mean. Error bars indicate SD. (**D**) qPCR analysis of CRISPR/Cas9-*KMT2A*r cells, non-*KMT2A*r patient (UPN1) and *KMT2A*r patient (UPN2) samples in contrast to control is shown. Each dot represents a sample. Horizontal lines represent the mean. Student’s *t* test was used: * *p* < 0.05. Error bars indicate SD. (**E**) Downregulated *FFAR2* expression in leukemic samples compared to other cancer types (data from oncomine.org). Boxes indicate the range from the 25th to the 75th percentiles; the horizontal lines represent the median; error bars indicate the range from 10th to 90th percentiles; the dots show the maximum and minimum values. (**F**) *FFAR2* expression in different patient leukemia entities compared to healthy controls (data from oncomine.org). Boxes indicate the range from 25th to 75th percentiles; the horizontal lines represent the median; error bars indicate the range from 10th to 90th percentiles; the dots show the maximum and minimum values. Student’s *t* test was used: * *p* < 0.05. (**G**) After 62 days of culture, *MLL-AF4* huCB and *MLL-AF4* huBM cells were treated with 5 µM of the FFAR2 antagonist GLPG0974 or vehicle (DMSO) and cell count was determined over time using Trypan blue. Student’s *t* test was used: * *p* < 0.05.

To gain more insight into the hampered transformation potential driven by *MLL-AF4* in huBM cells, we compared the expression profile from *MLL-AF4* huBM cells against all others and revealed 45 differentially expressed genes (Figure 5A). Strikingly, within these 45 genes, we uncovered *FFAR2*, also known as *GPR43* or *FFA2*, as the most downregulated in all other *KMT2A*r cells although it was less in *MLL-AF4* huBM cells indicating a possible important role in the *KMT2A*r transformation potential (Figure 5B,C). To further confirm the RNA-seq results, we performed qPCR with the samples submitted to RNA-seq and further CRISPR/Cas9-*KMT2A*r cells, *KMT2A*r patient (UPN1) and non-*KMT2A*r patient (UPN2) samples and revealed in all cases a significant downregulation of *FFAR2* in contrast to healthy controls but again a specifically less downregulation in *MLL-AF4* rearranged cells (Figure 5D). To elucidate the impact of *FFAR2* in cancer in general, we mined the literature and compared the expression level in different cancer entities. Strikingly, we discovered the lowest levels of *FFAR2* expression in breast cancer, prostate cancer and hematological diseases especially in leukemia (Figure 5E) [18]. This could be further confirmed by analyzing leukemia patient data of different entities, again showing very low levels of *FFAR2* in contrast to healthy controls (Figure 5F) [19]. Notably, after 62 days of cell culture, treatment with the FFAR2 antagonist GLPG0974 resulted in increased proliferation of *MLL-AF4* huBM cells whereas *MLL-AF4* huCB cells were not affected (Figure 5G) demonstrating the mechanistic role of FFAR2 in transforming *MLL-AF4* huBM cells [20].

Taken together, our data show that our CRISPR/Cas9-*KMT2A*r models are authentic by demonstrating common *KMT2A*r target gene expression that is not responsible for the immortalization. Moreover, we uncovered downregulation of *FFAR2* as a potential key player in the *KMT2A*r leukemogenesis.

## 3. Discussion

In this study we used CRISPR/Cas9 to generate *t*(9;11) and *t*(4;11) chromosomal translocations in primary human HSPCs derived from both huCB and huBM to unravel the differences on *KMT2A*r leukemia based on the fusion partner and the cell of origin, in which leukemia initiation occurs. Our models are based on patient-specific sequences and share not only genetical but also morphological, phenotypical and transcriptomic attributes of *KMT2A*r leukemias and hereby closely parallel the nature of the disease. Previously, we could demonstrate that by using CRISPR/Cas9 in huCB the creation of an authentic infant *KMT2A*r model based on endogenous oncogene activation was possible to unravel the pathogenesis of *KMT2Ar* leukemogenesis in vitro [14]. In this study, we successfully transferred our genetic tool to HSPCs derived from huBM and could again demonstrate the feasibility to generate both *MLL-AF4* and *MLL-AF9* translocations with high efficiency in an adult system. To our knowledge, this is the first demonstration that both the induction of *MLL* translocation and the outgrowth of pure *KMT2A*r cells in HSPCs derived from huBM is feasible. Interestingly, all generated cells except for *MLL-AF4* huBM cells showed unlimited growth potential in in vitro cultures. Although *MLL-AF4* huBM cells were similarly able to reach purity within 60 days and expressed *KMT2A*r target genes as a hallmark of *KMT2A*r leukemia, they succumbed to apoptosis in all performed experiments within around 80 days under myeloid culture conditions. This situation closely parallels human *KMT2A*r patients, as the portion of *MLL-AF4* myeloid leukemias in adults is negligible, whereas *MLL-AF4* leukemia is the most occurring lymphoid leukemia in infants [1]. One could argue that *MLL-AF4* cells become preleukemic in adults but full transformation to myeloid leukemia is not possible due to the cell of origin. Strikingly, prior to apoptosis our genome-engineered *MLL-AF4* huBM cells lost their CD9 surface expression as typical *KMT2A*r marker in in vitro cultures [16]. This indicates that CD9 loss is a potential early marker for detection of upcoming cell death upon targeted therapy. Further studies in *KMT2A*r patients during anti-cancer treatment are necessary to confirm this phenomenon. Our RNA-seq data of the CRISPR/Cas9-*KMT2A*r cells derived from huCB and huBM revealed a common *KMT2A*r signature including the overexpression of known common target genes like *MEIS1* and *HOXA9* that was irrespective of the fusion partner or cell of origin. This confirmed that our genome-engineered models based on patient sequences are authentic and that the observed differences are independent from the *KMT2A*r-typical target gene expression. Moreover, we could identify *FFAR2* as the most downregulated DEG in *MLL-AF9* huCB and huBM as well as *MLL-AF4* huCB cells. In contrast, *MLL-AF4* huBM cells failed to efficiently downregulate this gene indicating a major role for disease development. In addition, the fact that the FFAR2 antagonist GLPG0974 specifically favored the proliferation of *MLL-AF4* huBM cells underpins the essential role of FFAR2 in *KMT2A*r leukemogenesis [20]. Until now, the impact of *FFAR2* is only poorly understood in leukemia. It has been demonstrated that downregulation of *FFAR2* is necessary for leukemia survival in vitro and in vivo [21]. By re-analyzing publicly available datasets, we were able to assign *FFAR2* an important role in leukemia by demonstrating a significant downregulation in leukemic patients in contrast to different other tumor entities and healthy controls [18,19]. We could confirm this observation by performing qPCRs with our CRISPR/Cas9-*KMT2A*r models and patient samples. Interestingly, in colon cancer *FFAR2* acts as an epigenetic tumor suppressor since loss of *FFAR2* leads to high-level of H3K4me3 promoting colon carcinogenesis [22]. Importantly, leukemic stem cells (LSC) in *KMT2A*r leukemia display high levels of H3K4me3 and low levels of H3K79me2 thereby playing a crucial role in determining LSC fate [23]. Recently, a FFAR2 agonist with favorable pharmacokinetic properties has been developed allowing the targeting of FFAR2 as a new therapeutic strategy in *KMT2A*r leukemia [20].

In summary, our study highlights the feasibility of engineering chromosomal translocations at their endogenous loci in primary human HSPCs derived from both huCB and huBM to generate pure *KMT2A*r cells in a short period of time serving as innovative and authentic human leukemia model. Further, we provide robust data that *MLL-AF4* was unable to transform HSPCs derived from huBM cells under myeloid conditions indicating an important role of the fusion partner and the cell of origin. Downregulation of *FFAR2* seems to be mandatory for leukemia development and therefore could serve as therapeutic target in the treatment of poor prognosis *KMT2A*r leukemia.

## 4. Materials and Methods

### 4.1. Human CRISPR/Cas9-KMT2Ar Model and Patient Samples

CD34^+^ HSPCs were isolated (human CD34 MicroBead Kit UltraPure, Miltenyi, Bergisch Gladbach, Germany) either from fresh huCB obtained from the Department of Gynecology (IRB approval 751/2015BO2) or from huBM obtained from the Department of Hematology and Oncology of the University Hospital Tuebingen (IRB approval 309/2018BO2) and maintained in culture as previously described [14]. CRISPR/Cas9 was used to target patient-specific *MLL-AF4* and -*AF9* breakpoints for *KMT2A*r model induction and T7 endonuclease I assay (NEB, Ipswich, MA, USA) was performed to evaluate cutting efficiencies [14]. Rearrangements were identified via PCR (AccuPrime Pfx DNA Polymerase, Thermo Fisher Scientific, Waltham, MA, USA), reverse transcriptase (RT)-PCR, Fluorescence in situ hybridization (FISH, Cytocell *MLL* (*KMT2A*) Breakapart Probe, Cambridge, UK), karyotyping and Sanger sequencing (Seqlab, Goettingen, Germany) as previously described [14]. For compound treatment the FFAR2 antagonist GLPG0974 (Tocris, Bristol, UK) was prepared in a stock solution with DMSO [24,25,26,27]. After 62 days of culture *KMT2A*r huCB and huBM cells were subjected to treatment as indicated. Cells were retreated and reseeded at original density every second day. Cell counts were determined by staining with Trypan blue (Gibco, Thermo Fisher Scientific) using the Neubauer counting chamber.

Patient samples (UPN1, non-*KMT2A*r and UPN2, *KMT2A*r) were isolated from fresh peripheral blood obtained from the Department of Hematology and Oncology (IRB approval 137/2017BO2), frozen in RPMI 1640 medium supplemented with 20% filtered fetal bovine serum (FBS) (Merck-Millipore, Darmstadt, Germany) and 10% DMSO and thawed on demand.

### 4.2. Quantitative PCR (qPCR)

Total RNA was isolated (NucleoSpin RNA Kit, Macherey Nagel, Dueren, Germany), cDNA was generated (Thermo Fisher Scientific) and qPCR was performed for detection of *MEIS1*, *HOXA9* and *FFAR2* using 18S rRNA as housekeeper and employing the ddCT method as previously described [14]. The results were normalized on 18S rRNA and respective control cells were used as calibrator. Primers for *FFAR2* detection were: huFFAR2 FOR 5′CCCTCACGAGTTTTGGCTTC and huFFAR2 REV 5′GGAGCCACGTGCTGCAGTA.

### 4.3. May–Gruenwald–Giemsa Cytospin Staining

Cytospins were performed as previously described [14]. Images were collected using a Zeiss Primovert microscope with an ×40 objective and the Axiocam 105 color camera using ZEN 3.0 blue edition software (all Carl Zeiss AG, Oberkochen, Germany) at a resolution of 2560 × 1920 pixels.

### 4.4. Flow Cytometry

Analyses were performed using an LSR II flow cytometer (BD Biosciences, San Jose, CA, USA) and FACS DIVA software (BD Biosciences). For analyses, the following fluorochrome-conjugated monoclonal antibodies were used: CD34-APC (clone 4H11, eBioscience, San Diego, CA, USA), CD38-PE/Cy7 (clone HIT2, eBioscience), CD64-APC/Cy7 (clone 10.1, BioLegend, San Diego, CA, USA), CD33-BV421 (clone WM53, BioLegend), CD15-BV605 (clone W6D3, BioLegend, San Diego, CA, USA), CD117-BV711 (clone 104D2, BioLegend), CD14-Alexa Fluor 700 (clone HCD14, BioLegend) and CD9-PE (clone eBioSN4/SN4 C3-3A2, eBioscience). The analyses were pre-gated on single cells using forward scatter height (FSC-H) vs. forward scatter area (FSC-A) and subsequently on living cells by staining with Fixable Viability Dye eFluor 506 (eBioscience). Data were analyzed using FlowJo (TreeStar, V10, Ashland, OR, USA).

### 4.5. Cell Proliferation Analysis

Increasing amount of translocation positive cells was identified on DNA level via semi-quantitative PCR (AccuPrime Pfx DNA Polymerase, Thermo Fisher Scientific) of 100 ng genomic DNA with primers as previously described [14]. Proliferation of polyclonal cultures was determined by staining with Trypan blue (Thermo Fisher Scientific) and total cell count was calculated over a period of 120 days. Cell viability was determined by flow cytometry using Fixable Viability Dye eFluor 506 (eBioscience).

### 4.6. RNA Sequencing and Gene Expression Analyses

RNA was isolated (NucleoSpin RNA Kit, Macherey Nagel) and quality assessment was carried out by NanoDrop (Thermo Fisher Scientific) and Bioanalyzer measurements (Agilent, Santa Clara, CA, USA).

Sequencing of the Quantseq 3′ mRNA libraries was performed on a NextSeq 500 platform (Illumina Inc., San Diego, CA, USA) at a resolution of ~6–11 mio single end reads per sample and 101 bp in length. Reads were trimmed and cleaned up from contaminating adapters, polyA read through events, and low quality tails with BBDuk of the BBMap [28] tools suite (v38.67) using the following parameters: (k=13 ktrim=r useshortkmers=t mink=5 qtrim=r trimq=10 minlength=20) as well as employing the polyA and truseq_rna k-mer resources. Quality of the cleaned fastq files was assessed using FastQC (v0.11.4) [29] before aligning reads with STAR (v2.7.0a) [30] against the Ensembl *H. sapiens* genome v95 using the following parameters: (--outFilterType BySJout --outFilterMultimapNmax 20 --alignSJoverhangMin 8 --alignSJDBoverhangMin 1 --outFilterMismatchNmax 999 --outFilterMismatchNoverLmax 0.6 --alignIntronMin 20 --outSAMattributes NH HI NM M). Read counts for all genes were obtained using the *featureCounts* function of Rsubread (v2.0.0) with (GTF.featureType=“exon” GTF.attrType=“gene_id” useMetaFeatures=TRUE strandSpecific=1) and DESeq2 (v1.26) [31]. Transcripts covered with <1 read were excluded from subsequent analyses leaving 21,978 genes for determining differential expression. Significance thresholds were set to |log2 FC| ≥ 1 and BH-adjusted *p*-value ≤ 0.01. Surrogate variable analysis (sva, v3.26.0) [32] was used to minimize unwanted variation between samples.

### 4.7. Statistical Analyses

To summarize pooled data of independent experiments the mean was calculated and standard deviation (SD) was used to describe the variability. Student’s *t* test was used for statistical analysis and *p* < 0.05 was considered statistically significant.

### 4.8. Data Sharing Statement

For original data, please contact corina.schneidawind@med.uni-tuebingen.de. Raw sequencing files and count data are available through Gene Expression Omnibus (GEO) under accession number GSE148714.

## 5. Conclusions

In this study, we used CRISPR/Cas9 to introduce translocations of the *MLL (KMT2A)* and *AF4* or *AF9* genes under physiologic promotors in both huCB and huBM cells. All genome-engineered cells faithfully mimic the genuine nature of the disease by sharing morphological, phenotypical and transcriptomic attributes of *KMT2A*r leukemias therefore constituting an innovative and authentic human model of *KMT2A*r leukemia. However, the oncogene *MLL-AF4* only transformed cells derived from infant cells demonstrating a major role of the fusion partner and the cell of origin in leukemia development. Finally, we uncovered intrinsic properties like an absent downregulation of free fatty acid receptor 2 (*FFAR2*), an epigenetic regulator, possibly responsible for this phenomenon.
